# Knowledge about the emergency management of dental injuries among field hockey coaches

**DOI:** 10.1111/edt.12774

**Published:** 2022-07-08

**Authors:** Kirsten E. van Vliet, Henk S. Brand, Frank Lobbezoo, Jan de Lange

**Affiliations:** ^1^ Department of Oral and Maxillofacial Surgery, Amsterdam UMC University of Amsterdam/Academic Centre for Dentistry (ACTA) Amsterdam The Netherlands; ^2^ Department of Oral Biochemistry, Academic Centre for Dentistry (ACTA) University of Amsterdam and Vrije Universiteit Amsterdam Amsterdam The Netherlands; ^3^ Department of Orofacial Pain and Dysfunction, Academic Centre for Dentistry Amsterdam (ACTA) University of Amsterdam and Vrije Universiteit Amsterdam Amsterdam The Netherlands

**Keywords:** coaches, dental injury, field hockey, management, tooth avulsion

## Abstract

**Background/Aim:**

Field hockey is frequently associated with oro‐dental injuries. In such acute situations, appropriate management of the injury by coaches can contribute to a better clinical outcome and prognosis. Therefore, the aim of this study was to investigate the knowledge of hockey coaches in the Netherlands about the emergency management of dental injuries.

**Material and Methods:**

A 25‐item questionnaire about the prevalence and management of oro‐dental injuries was distributed amongst hockey coaches in the Netherlands. A sub‐analysis was done regarding the knowledge on the management of dental injuries by using a 5‐point scoring system.

**Results:**

Two hundred and six hockey coaches participated in this survey. A small majority (53%) of the coaches knew the treatment of choice in the event of a permanent tooth injury. The median score of coaches' knowledge regarding management of oro‐dental injuries was 3.0 (2.0–4.0) points. Coaches with a (para)medical training/occupation, and coaches with >10 years of experience had significantly higher scores compared with the other groups (*p* < .01 and *p* = .03, respectively).

**Conclusions:**

The knowledge level of hockey coaches in the Netherlands about oro‐dental injuries needs to be improved, as many coaches are not aware of the appropriate first aid measures.

## 1 INTRODUCTION

Oro‐dental injuries frequently occur in ball and contact sports, especially in a sport such as field hockey. A systematic review on injuries during field hockey concluded that head injuries (2%–50%) are the most common type of injury after lower limb injuries (13%–77%).^1^ To prevent hockey players from oro‐dental injury, players are advised to wear mouthguards or face shields. Furthermore, hockey rules have been changed over time in order to make the game safer and to reduce the risk of having oro‐dental injuries.^2^ However, despite these efforts, oro‐dental injuries still frequently occur in field hockey players.^3^, ^4^ It is therefore of importance that hockey players and their coaches have knowledge of first aid management of dental injuries which may occur while playing hockey. If dental injuries are not properly treated, they can result in long‐term dental problems with subsequent emotional problems associated with facial injuries.^5^ The correct management of an acute avulsed tooth is described in the guidelines of the International Association of Dental Traumatology (IADT).^6^ These guidelines state that care should be taken to avoid touching the root of the tooth and when dirty, to clean the tooth with milk, saline or saliva. The tooth should be kept moist and the dry period should be kept to a minimum. Immediate replantation of an avulsed permanent tooth is the recommended treatment.^6^, ^7^, ^8^

Several studies have explored the knowledge of athletes and school teachers about dental injuries, and they have also investigated the effect of additional education.^9^, ^10^, ^11^, ^12^, ^13^, ^14^, ^15^, ^16^, ^17^, ^18^, ^19^, ^20^ For example, Ivanda et al. examined 803 full‐time working elementary school teachers regarding their knowledge of oro‐dental injuries.^9^ They found an overall mean score of 6.6 ± 2.5 points, out of a maximum score of 14 points and concluded that there was a lack of knowledge among school teachers about the management of dental injuries.^9^ Karande et al. investigated the awareness of school teachers in India on prevention and management of dental injuries in school children.^10^ They concluded that teachers had a serious lack of knowledge about the proper treatment in the event of a dental injury. Three months after education by dentists, the knowledge of the teachers about emergency management had improved significantly.^10^ Another study among sportsmen and women showed an overall lack of knowledge with regard to tooth avulsion.^13^ Less than half (42.6%) would visit a dentist for treatment, 51.7% would replant the avulsed tooth, and only 6.5% would place the avulsed tooth in milk.^13^ The authors of the latter study strongly recommend information campaigns to improve the immediate emergency treatment of tooth avulsion.

The above studies demonstrate a lack of sufficient knowledge about oro‐dental injuries in sport, and education can improve the management and outcome of oro‐dental injuries. However, no studies have reported the knowledge about the management of oro‐dental injuries among Dutch field hockey coaches. Since field hockey is one of the most popular sports in the Netherlands (with over 240,000 members in 2020^21^), this information could be important to determine whether the outcome corresponds with the lack of knowledge found in the literature. Therefore, the aim of this study was to investigate the current knowledge of field hockey coaches in the Netherlands regarding the management of oro‐dental injuries.

## 2 MATERIALS AND METHODS

A 25‐item questionnaire about the management of oro‐dental injuries was developed. The questionnaire contained the following four consecutive parts: personal characteristics of the coaches, experienced oro‐dental injuries during coaching, management of oro‐dental injuries, and previous education about oro‐dental injuries. The knowledge of the coaches regarding the management of dental injuries in part 3 was analyzed with 5 questions, using a 2‐point scoring system. A correct answer scored 1 point, and an incorrect answer scored 0 points. Some questions had several correct answers. Regardless of which correct answer was chosen, 1 point was provided for these questions (Table 1). Hence, coaches could receive a maximum score of 5 points.

**TABLE 1:** Scoring system regarding the coaches' knowledge about the management of oro‐dental injuries

Question	Answer	Points
What would you do if a primary tooth is avulsed?	Discard/not search for the tooth	1
	Rinse the tooth with water	0
	Wrap the tooth in a clean handkerchief/gauze	0
	Rinse the tooth with water and wrap in a clean handkerchief/gauze	0
	Preserve the tooth in milk	0
	Preserve the tooth in a saline solution	0
	Preserve the tooth in a disinfectant	0
	Preserve in the mouth/saliva	0
What would you do if a permanent tooth is avulsed?	Discard/not search for the tooth	0
	Rinse the tooth with water	0
	Wrap the tooth in a clean handkerchief/gauze	0
	Rinse the tooth with water and wrap in a clean handkerchief/gauze	0
	Preserve the tooth in milk	1
	Preserve the tooth in a saline solution	1
	Preserve the tooth in a disinfectant	0
	Preserve in the mouth/saliva	1
What is the most suitable medium to store an avulsed tooth?	Water	0
	Milk	1
	Saliva	1
	Isotonic sports drink	0
	Saline solution	1
	Alcohol/Disinfectant	0
	None, it is best to keep the tooth dry	0
If a part of the tooth is broken, would you search the field for the fragment?	Yes	1
	No	0
Do you think a tooth fragment can be reattached?	Yes	1
	No	0

The inclusion criteria were that the respondents were currently coaching for at least 1 year, employed or on a voluntary base, in Dutch field hockey. Coaches with experience <1 year were excluded. A preliminary version of the questionnaire was tested on five field hockey coaches. Their feedback led to some small adjustments of the questionnaire. The items of the final version of the questionnaire were entered into Formdesk, a web‐based survey tool (Innovero Software Solutions BV). A link to the questionnaire was placed on an online platform for field hockey coaches in the Netherlands, in the period from October 26 to December 9, 2020. At that time, this platform had 4605 members. The minimum required sample size (*n* = 355) was calculated from this number of members (*n* = 4605) with a 95% confidence interval, 5% margin of error, and a population proportion of 50%. The questionnaire reached 2385 members of the online platform. A final total of 206 questionnaires were included in this study. Participants could complete the questionnaire only once.

This study was approved by the Ethics Committee of The Academic Centre for Dentistry in Amsterdam (ACTA) (#2020276) and was performed in accordance with the ethical principles stated in the Declaration of Helsinki. Participation was on a voluntary basis and the answers to the questionnaire were processed completely anonymously. Informed consent was automatically obtained when the participant began to complete the online survey.

Statistical analyses were performed using SPSS, v. 26 (IBM SPSS Statistics for Windows). Standard descriptive statistics were used to describe the sample. As the Kolmogorov–Smirnov test showed that the dependent variable was not normally distributed, further statistical analysis was conducted using the Kruskal–Wallis tests and Mann–Whitney *U* test. As significance level, alpha was set at 0.05.

## 3 RESULTS

Demographic and professional characteristics of the respondents are presented in Table 2. The group respondents with medical or paramedical training/occupation comprised 0 dentists, 3 physicians, 2 nurses, 2 dental hygienists, 3 physiotherapists, 10 medical students, 7 subjects who had completed a first aid course, and 9 had another paramedic occupation. Almost all coaches (99%) encouraged players to wear a mouthguard, and 90% reported verifying whether their players were wearing a mouthguard. Most coaches (92%) believed that mouthguards are effective in the prevention of oro‐dental injuries. Eighty‐two percent of the coaches had experienced an oro‐dental injury to a player being coached by them. At the time of the injury, 81% of the coaches contacted the parents of the player involved, sometimes combined with consulting the (team) physician, 15% contacted the emergency service of the dentist or oral and maxillofacial surgeon, and 4% did not contact anyone.

**TABLE 2:** Characteristics of field hockey coaches who participated in this study

Description	*N* = 206 (100%)
Gender	
Male	99 (48%)
Female	106 (52%)
Not reported	1 (<1%)
Mean age ± SD (in years)	32 ± 14
Medical or paramedical training/occupation	36 (18%)
Currently or former hockey player	188 (91%)
Oro‐dental injury during own hockey career^a^	93 (50%)
Did you wear a mouthguard^a^	152 (81%)
Coached players	
Junior	141 (68%)
Senior	6 (3%)
Both	59 (29%)
Coach experience	
<5 years	74 (36%)
5–10 years	85 (41%)
>10 years	47 (23%)

^a^ Based on the data from coaches who are currently or former hockey players themselves(*n* = 186).

Concerning the preservation of an avulsed primary tooth, 84 coaches (41%) would preserve the tooth in a clean handkerchief/dry gauze, while 57 (28%) would clean the tooth with water first and subsequently preserve it in a handkerchief/gauze. Forty‐six coaches (22%) would preserve the tooth in milk, 1 (<1%) in saline solution, 1 (<1%) in a disinfectant, and 3 (2%) in the mouth/saliva. Fourteen coaches (7%) would discard the tooth or not search for it. This means that only 7% of the coaches answered this question correctly.

**FIGURE 1:**
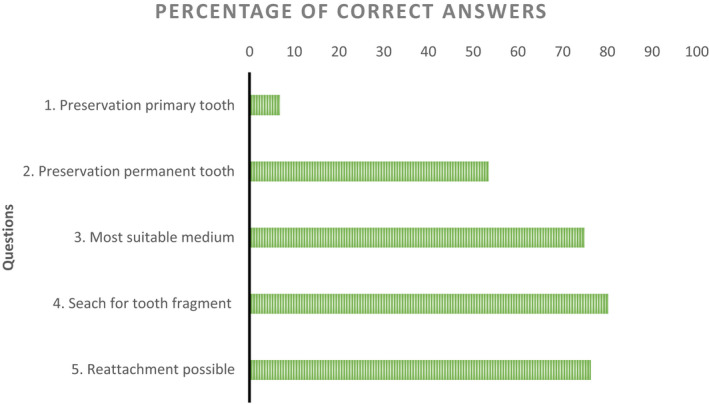
Percentage of correct answers from the coaches on the five questions about the preservation of avulsed teeth, and re‐attachment of tooth fragments

Concerning the preservation of an avulsed permanent tooth, 48 coaches (23%) would preserve the tooth in a clean handkerchief/dry gauze, 1 (<1%) would clean the tooth with water, and 44 (21%) would clean the tooth with water first and then preserve it in a handkerchief/gauze. Eighty‐nine coaches (43%) would preserve the tooth in milk, 4 (2%) in saline solution, 3 (2%) in a disinfectant, and 17 (8%) in the mouth/saliva. This indicates that 53% of the coaches chose a correct option for this question. With regard to the most suitable medium to preserve an avulsed tooth, 77 coaches (37%) reported they would store the avulsed tooth in milk, 70 (34%) in the mouth/saliva, 25 (12%) in water, 7 (3%) in saline solution, 1 (<1%) in an isotonic sports drink, and 1 (<1%) in a disinfectant. Twenty‐five coaches (12%) thought that the avulsed tooth should be stored dry. Therefore, 75% of the coaches answered this question correctly by preserving the avulsed permanent tooth in milk, saline, or in the mouth/saliva. In the event that only a fragment of a tooth is missing, 80% of the coaches answered correctly that they would look for it. Seventy‐six percent of the coaches thought correctly that a dentist can re‐attach a fractured tooth fragment (Figure 1).

The median score for the correct management of oro‐dental injuries among all coaches was 3.0 (range 2.0–4.0) points. The median scores of correct answers, stratified according to gender, (para)medical training/occupation, coaching experience, education received, and need for education are shown in Table 3. The median scores of coaches with a (para)medical training/occupation were significantly higher compared with coaches without (para)medical training/occupation. Coaching experience also had a significant effect on the number of correct answers. The median score of coaches who had been educated in the past was not significantly different from those who had not received education on this topic. Two‐thirds of the coaches (67%) would like to receive education regarding the management of oro‐dental injuries that they may face while coaching. These coaches mentioned several ways as to how this information should be disseminated: 29% preferred an informative leaflet, 29% through the website of the Royal Dutch Hockey Association (KNHB), 23% thought it should be included in the curriculum of hockey coaches, 13% preferred an online course, and 7% wanted multiple educational platforms.

**TABLE 3:** Median scores of correct answers, stratified according to gender, (para)medical training/occupation, coaching experience, education received, and the need for education

Description	Median score (25%–75%)	*p*‐value
Gender		
Male (*n* = 99)	3.0 (2.0–4.0)	.89
Female (*n* = 106)	3.0 (2.0–4.0)	
(Para) medical training/occupation		
Yes (*n* = 36)	4.0 (3.0–4.0)	<.01
No (*n* = 170)	3.0 (2.0–4.0)	
Experience as a coach		
<5 years (*n* = 74)	3.0 (2.0–4.0)	.03*
5–10 years (*n* = 85)	3.0 (2.0–4.0)	
>10 years (*n* = 47)	4.0 (3.0–4.0)	
Education on orofacial injuries in the past		
Yes (*n* = 26)	4.0 (2.0–4.0)	.12
No (*n* = 180)	3.0 (2.0–4.0)	
Need for future education		
Yes (*n* = 137)	3.0 (2.0–4.0)	.72
No (*n* = 69)	3.0 (2.0–4.0)	

* Sub‐analysis between groups showed a significant difference only between the coaches with <5 years and the coaches with >10 years of experience: *p* = .03).

## 4 DISCUSSION

This study investigated the current knowledge of field hockey coaches in the Netherlands regarding the management of oro‐dental injuries since the literature shows a lack of knowledge among all other sports coaches and teachers. In this study, a large majority of the field hockey coaches had witnessed an oro‐dental injury during their career as a hockey coach. However, their knowledge regarding the correct management of oro‐dental injuries is insufficient. The median score for the correct management of oro‐dental injuries among all coaches was 3.0 points, out of a maximum score of 5 points. Coaches with a (para)medical training or occupation, and coaches with >10 years of experience had significantly higher scores. This is in accordance with the results of a study by Sepet et al.^12^ In their survey of 359 players of different sports in Turkey, participants with more than 5 years experience had significantly more knowledge about dental emergency procedures and mouthguards compared with the less‐experienced participants.

Recently, Bazina et al. performed a similar survey of the knowledge about the management of dental injuries among 51 water polo coaches in Croatia.^22^ They reported a poor knowledge of water polo coaches with regard to oro‐facial injuries with an overall mean score of 1.61 ± 1.16 points out of a maximum score of 6 points. One water polo coach (2%) would preserve an avulsed tooth in a saline solution, which is comparable with the percentage of hockey coaches in the present study. However, not a single water polo coach would preserve an avulsed tooth in milk while in the present study 43% of the hockey coaches would preserve avulsed teeth in milk. The number of water polo coaches who believed that a fractured tooth fragment could be re‐attached by a dentist was also lower than the number of hockey coaches (57% vs. 76%). Overall, these results suggest that the knowledge of field hockey coaches about the management of oro‐dental injuries in the Netherlands is slightly better than the knowledge of water polo coaches in Croatia.

Notwithstanding the above, with less than half of the questions correctly answered by the coaches in this study, there is still room for improvement since tooth injury is a real emergency situation and appropriate treatment at the site of the accident improves the chances for optimal recovery.^6^ In general, avulsed teeth have the worst prognosis and they account for 1%–3% of all dental injuries. Immediate correct preservation of the tooth and fast replantation is crucial for tooth survival.^5^, ^23^ Adequate education of athletic personnel can reduce morbidity. This is recognized by The Council of Clinical Affairs of the American Academy of Pediatric Dentistry, which recommends coaches consult with a dentist who has expertise in oro‐dental injuries at the start of a sporting season. The Council also highly recommends dentists, who have a professional responsibility, to self‐educate themselves about sport‐related dental injuries and to play an active role in educating the public regarding sport‐related dental injuries.^24^

In this study, coaches with a (para)medical training/occupation had significantly higher scores compared with the other coaches, but a similar effect was not seen for coaches who reported that they had received education on oro‐dental injuries. This could indicate a low quality of the previous education and implies that improvement is needed. Since these results suggest that (para)medical training is more effective, it seems reasonable to involve (para)medical professionals in determining the content of courses about the management of oro‐dental injuries for hockey coaches. The present study has also shown that a large majority of field hockey coaches are receptive to receiving additional education regarding the management of oro‐dental injuries. The hockey coaches mostly preferred education through an informative leaflet or the website of the Royal Dutch Hockey Association (KNHB). However, previous research has shown that actively engaging people in a topic is the most effective way of receiving information and learning, rather than passively following a presentation.^25^ Providing information visually, for example, through e‐learning with videos, results in the information offered being memorized for a longer period of time than providing the same information without videos.^26^, ^27^ This suggests that videos should be part of the course material for field hockey coaches. Also, the mobile app, called ToothSOS, developed by the International Association of Dental Traumatology (IADT) contains visual information and a step‐by‐step plan for each type of dental injury. A recent study by Duruk et al. reported that this App is an effective training tool to improve knowledge among non‐dentists.^28^

The present study has several limitations. Firstly, a possible response bias could have affected the results. Participating coaches may have had a certain interest in oro‐dental injuries. Also, coaches must be active in the online member platform where the questionnaire was posted. Therefore, the results could be an over‐estimation of the knowledge and this points towards a need for even more education. Although the respondents had been instructed not to look up the correct answers, it is possible that participants had searched for the correct answer which would also result in an over‐estimation of their knowledge. Also, participants could only choose one (best) answer, while some questions had multiple correct answers, which could have led to confusion.

Irrespective of these limitations, this study provides some interesting insights into the knowledge of field hockey coaches on the management of oro‐dental injuries. On average, coaches of the second most popular team sport in the Netherlands have limited knowledge about the adequate treatment of oro‐dental injuries. The information obtained in the present study can be used when developing educational programs for field hockey coaches. This can increase the awareness of the hockey coaches and increase the chances that field hockey players will receive the proper first aid treatment after oro‐dental trauma.

## 5 CONCLUSION

A large group of field hockey coaches in the Netherlands is not aware of the appropriate first aid action that is warranted in the event of an oro‐dental injury. Considering that coaches can play a crucial role in the acute management of an injury, special attention should be given to further education of coaches on this topic.

## AUTHOR CONTRIBUTIONS

KvV and HB were involved in the study design and the development of the digital questionnaire. HB distributed and collected the data. KvV performed the statistical analysis. KvV, HB, FL and JdL contributed to the writing of this paper and were involved in the interpretation of the results and the discussion. All authors read and approved the final version of the manuscript.

## CONFLICT OF INTEREST

The authors declare no conflict of interest.

## Data Availability

The data that support the findings of this study are available from the corresponding author upon reasonable request.
